# Multiple factors contribute to female dominance in migratory bioflows

**DOI:** 10.1098/rsob.240235

**Published:** 2025-02-12

**Authors:** Toby D. Doyle, Oliver M. Poole, Jaimie Christopher Barnes, Will Leo S. Hawkes, Eva Jimenez Guri, Karl R. Wotton

**Affiliations:** ^1^ Centre for Ecology and Conservation, University of Exeter, Cornwall Campus, Penryn, UK; ^2^ Swiss Ornithological Institute, Seerose 1, Sempach CH-6204, Switzerland

**Keywords:** sex bias, genetics of migration, insect migration, migratory hoverflies, Syrphidae, *Episyrphus balteatus*

## Abstract

Migration is a widely observed phenomenon supported by morphological, physiological and behavioural traits that vary with season and sex in many species. Recently, the genetic components underpinning migration in the marmalade hoverfly (Diptera: Syrphidae) have been unpacked through detection of differentially expressed genes between migrant and non-migrant females. Males also migrate, but changing sex ratios during autumn migration, from around 50% female in northern Europe to around 90% in southern Europe, suggests males are poor long-distance fliers. To elucidate the mechanisms underpinning this sex difference, we performed morphological, physiological and transcriptomic characterization of actively migrating females and males. Both sexes show similar physiological adaptations including hyperphagia and starvation resistance, but females display higher tolerance to cold, have lower wing loading values and display a greater flight capacity. In addition, females modulate the expression of genes involved in immunity, hypoxia and longevity while suppressing hormonal pathways involved in maintaining reproductive diapause. These traits contribute to the success of female migrants and underlie the diminishing pool of males, influencing population dynamics across huge geographic areas and through the whole migratory and overwintering period.

**Figure:**
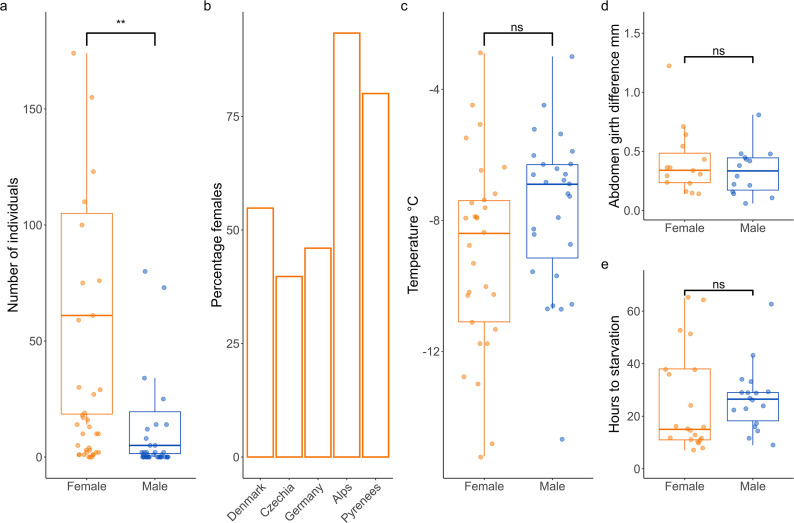

## 1. Introduction

Migration is a diverse and widely observed phenomenon encompassing a variety of taxa from large birds to small flying insects and occurs on all continents of the world. Migratory events are primarily driven through changes in resources leading to the movement to sites with more favourable conditions outside the current range [1]. The traits necessary to sustain long distance travel are far-reaching and complex, including morphological, physiological and behavioural adaptations that have evolved concurrently giving rise to a migratory syndrome [2]. The genetics of the migratory syndrome are increasingly being unpacked and this is particularly the case in the study of insect migration where populations can be sampled and sequenced with relative ease, with examples ranging from the globe skimmer dragonfly (*Pantala flavescens*), monarch butterfly (*Danus plexippus*), the marmalade hoverfly (*Episyrphus balteatus*) and pest moth species such as the cotton bollworm (*Helicoverpa armigera*) [3–6]. These species are part of an economically and biologically important, worldwide migratory assemblage that distributes beneficial ecosystem services such as pollination and pest control and sustains human livelihoods, while also in some cases being detrimental through the spread of pest populations and disease [7–12]. Understanding these systems in an age of range expansion in some species and decline in others, often linked to a changing climate, is critical [13,14].

Despite many historical reports of hoverfly migration, the family has only recently emerged as a powerful model for insect migration. Migration in hoverflies is widespread and found in all subfamilies of the Syrphidae family with an estimate 212 migratory hoverfly species worldwide [15]. Hoverflies are known to migrate on all continents except for Antarctica, including flying to incredible altitudes in Nepal [16] and forming dense migratory flows in North America [17,18]. However, it is best characterized in Europe with many migratory flyways elucidated in recent years [19–24]. During migration hoverflies can cover hundreds of kilometres in a day [25] with many moving thousands of kilometres to complete their migration [26–28]. The mechanisms underpinning the migratory phenotypes are complex and wide-ranging including the use of a time-compensated sun compass as a navigational mechanism [29] or the induction of an energy conserving state to reduce non-essential energy expenditure [30]. During autumn migration females are in a state of reproductive diapause with resources thought to be redirected to flight, fat storage and increased lifespan with only females surviving until the spring [31]. Interestingly, these females also appear to be mated with sperm stored in their spermathecae [32]. Recently Doyle *et al.* [6] unpacked the genetic mechanisms of migration through a genome-wide transcriptomic comparison of actively migrating *E. balteatus* identifying a suite of differentially expressed migratory associated genes with roles in metabolism, muscle structure and function, hormonal regulation, immunity, longevity and reproductive diapause. Remarkably, sex biases favouring females have been reported during southward migration in many migratory species moving along the western European flyway [22,33,34] but the reasons for this bias remain unexplained.

Bias in sex ratios are common in migrants, with most research focusing on vertebrates [2] where sex ratios can alter drastically with a variety of behaviours and mechanisms employed to achieve reproductive success and survival. For example, many bird species exhibit protandrous behaviour, the earlier arrival of male birds at breeding sites to hold territory [35], while in some bat species females fly further and do so while pregnant [36]. During insect migration, sex-specific life histories and migration strategies also induce sex biases, such as the earlier spring departure of male monarch butterflies [37] or the earlier emergence of female rice leafroller moths *Cnaphalocrocis medinalis* [38]. These differences are thought to lead to biases in sex ratios during migratory events [38], in extreme cases such as the pine shoot moth *Rhyacionia buoliana*, males do not migrate at all, but mate with the females prior to migration to ensure reproductive success [39]. Despite the prevalence of sex biases in migrants, the underlying causes and genetic bases have seldom been investigated [40]. Here we leverage the hoverfly system to explore the mechanisms behind female biased sex ratios with a series of morphological, physiological and transcriptomic investigations that compare actively migrating female and male migrants caught in the Pyrenean mountain pass of Bujaruelo. This mountain pass efficiently funnels migrating insects during their southward migration in autumn which, in the right environmental conditions, creates an area of dense insect movement. We hypothesize that females dominate this migratory bioflow owing to a variety of traits such as enhanced flight capabilities and resistance to starvation [41,42] that serve to reduce their overall mortality as compared with males during migration.

## 2. Methods

### 2.1. Location

We carried out our investigation into migratory insects at the Pyrenean Mountain pass of Puerto de Bujaruelo, Spain (42.7038793 N, −0.0641454 W). Permission to conduct observation and collect samples was obtained from the Parc National des Pyrénées (France, authorization numbers: 2018-9 and 2023-158) and the Gobierno de Aragon (Spain, authorization numbers: 500201/24/2018/06141, 500201/24/2019/02174 and 500201/24/2021/01722). Insect trapping took place over four years during autumn with samples taken either by hand net or using a ‘mini-Heligoland trap’ or a malaise ‘migration’ trap (see below for details).

### 2.2. Fly wing measurements and sex ratios

Insect samples were collected over 4 years of southward autumn migration using a BugDorm ez-Migration Trap II. This trap was set up during autumn field seasons running from 1 September until mid-October over 2018−2021. Insect samples were collected daily, identified using a Lecia dissection microscope and stored in 70% ethanol. The sex ratio and wing measurements of migrants was determined using 17 mass migration events (as defined by [19]) across 4 years, 2018, 2019, 2021 and 2023 (2020 excluded owing to limited *E. balteatus* migration), in addition we used wing length, width, area and aspect ratio data from Pyrenees hover flies caught in the 2023 season for other experiments, details below, with males determined by their holoptic eyes. To add a comparator for wing measurements we used our lab reared stocks (reared in summer like conditions 80% RH, L16.55 : D7.45, L20°C : D15°C) and wild-type summer caught individuals by the Natural History Museum (London, UK) (see electronic supplementary material, file S1). Intact flies were separated into individual tubes and dried for 48 h at 55°C to obtain the dry weight, using a Sartorius Cubis Micro Balance. The left wing from each fly was removed and placed between a microscope slide and coverslip with one drop of 50% glycerol. Slides were calibrated and imaged, then wing length (base of 2nd basal cell to R4+5 wing tip) and width (subcostal vein to junction of cell Cu1 and cell 2a) taken using Leica Acquire LAS EZ software at 2.5× magnification (electronic supplementary material, figure S1). Wing area was calculated using a Nikon Ds-Ri2 microscope at 2× magnification, slides were calibrated and imaged, then area was calculated using the NIS-Elements AR software. Only fully intact left wings were measured for the wing area, this initial area was then doubled to provide the wing area for the whole fly. Wing load was calculated by dividing dry body mass by wing area. The wing aspect ratio was calculated by wing length divided by wing width [43]. Analysis of the data was carried out in R version 4.3.0 [44]. Linear models were constructed using backwards stepwise model selection beginning with a model including all terms (sex and dry weight). Using the drop1 function, a single term was removed at a time and the result compared with the previous model to assess significance of the term. *Emmeans* was used as a post hoc pairwise comparison function [45]. Body condition could not be assessed due to sample having been stored in 70% ethanol.

### 2.3. Physiological adaptation experiments—cold tolerance, hyperphagia and starvation

For all physiological experiments, actively migrating male and female *E. balteatus* were caught in the mountain pass using an adapted Heligoland style insect trap constructed from garden mesh (7 × 7 mm diameter openings) with a 2 m opening which funnelled insects into a large insect cage (Watkins & Doncaster pop-up cage 90 × 60 × 60 cm). These insects were removed from the pass and placed into smaller insect cages with access to 60% organic honey water and ground organic pollen, except for starvation and hyperphagia experiments. All flies were placed into experiments within 4 h of being caught on the mountain pass. Body condition for cold tolerance (size of abdomen) was recorded by measuring the percentage of abdomen made up by the ventral clear section of the abdomen from a profile view, broadly following [41] for hyperphagia and starvation experiments body condition was not included as all samples were deemed to be medium condition. Analysis of the data was carried out in R version 4.3.0 [44]. To analyse cold tolerance, hyperphagia and starvation experiments linear models were constructed using backwards stepwise model selection, beginning with a model including all terms (body condition and sex). Using the drop1 function, a single term was removed at a time and the result compared with the previous model to assess significance of the term. Dry weight was not determined for these experimental samples.

### 2.4. Cold tolerance

To determine the cold tolerance of individual flies we used the super cooling point (SCP), a proxy of cold tolerance, using a well-established protocol whereby just before an organism completely freezes there is a sudden spike in temperature [46]. Individual flies were connected to electrodes with a small amount of petroleum jelly and placed into 1.5 ml tubes held in place with a small amount of packed cotton wool. Electrodes were connected to a Thermosense BTM-4208DSD 12 channel temperature logger, placed into a domestic freezer and allowed to freeze. The cold tolerance limit of an individual was deemed to be the lowest temperature reached before a sudden spike in temperature (SCP).

### 2.5. Hyperphagia

It has previously been observed that migratory hoverflies allowed to feed ad libitum develop distended abdomens suggestive of hyperphagia [41]. To measure this distension, we measured male and female abdominal girth with digital callipers prior to feeding. Measured flies were then placed into 300 ml pots with access to organic ground pollen and 60% organic honey water and left for 48 h in natural light settings and temperatures (~L12:D12, ~18°C) to feed ad libitum. After 48 h the flies were measured again to determine changes in abdomen distension by the same person, to avoid observational bias.

### 2.6. Starvation resistance

Unfed flies were placed into four 30 × 30 cm BugDorms, separated by sex with 9−11 individuals per cage. A Wileyfox camera phone was placed on a retort stand above each cage and set to take a photo every hour through the clear plastic cage tops. Flies were left in a room subject to overhead artificial multi-LED light (Radion XR15 G4, EcoTech Marine at 95W) and a constant temperature of approximately 21°C. Upon the death of the last fly the image data was analysed to determine the hour at which each fly died.

### 2.7. Flight mill

Newly captured actively migrating flies were stored in 30 × 30 cm BugDorms for 1–3 days and fed ad libitum as per above. Insects were stored inside at 18–23°C with up to 15 individuals per container and exposed to natural light cycles. Flies were flown inside within naturally active periods of their circadian cycles beginning between 08.00 and 11.00, using an overhead artificial multi-LED light to simulate the midday sun and maintained in temperature ranges typical of Pyrenean early autumn (18–23°C). Individual flies were tethered to an L-shaped pin by the thorax using a UV adhesive (Bondic). This pin was fixed to the suspended arm of the flight mill to allow recording of forward and backward movement using dual-channel photointerruptors connected to a Raspberry Pi (following [41]). A 4 h recording period began immediately after forced flight was initiated by sudden removal of an object under the fly’s grip. After milling, all flies were imaged under a light microscope for body condition categorization, categorized as either thin (0–34.9%), medium (35–59.9%) or fat (60–100%) Flight mill data was processed in Python [41] and analysed in R version 4.3.0 [44] using the *glmmTMB* package [47]. We used generalized linear mixed effects models (GLMMs) to test the effects of sex, body condition and their interaction on flight performance metrics. Distance, acceleration, flight time, longest flight, number of flights, mean flight duration, and mean and maximum speed were fitted as the response variables in separate models. Two flight mills, each with four operating arms were used to collect the data and thus ‘flight mill’ and ‘mill arm’ were fitted as nested random effects to account for subtle functional differences between mills and their operating arms. Backwards stepwise model selection using the drop1 function allowed determination of the best fitting model for each flight metric. Best fitting models were analysed against the previous model using a chi-squared test.

### 2.8. Transcriptome sequencing and expression analysis

Female and male migrant flies were collected from the mountain pass during September–October 2018 (electronic supplementary material, table S1), these samples were all collected in the afternoon on the same day except for two females which were collected on a different day. These samples are all drawn from a single European panmictic population. All samples were snap frozen in the field using a dry shipper and stored at −80°C. Total RNA was extracted using standard TRIzol protocol (Invitrogen) and treated with DNAse using TURBO DNAse (Invitrogen) following the manufacturer’s guidelines. Quality and quantity of total RNA was assessed using Qubit BR RNA quantification kit (Invitrogen), Nanodrop (Invitrogen) and Bioanalyser 2100 (Agilent). In total, 20 RNA samples (10 female and 10 male) were sent to Exeter sequencing centre for library construction and RNA-seq. TruSeq stranded mRNA-seq libraries were prepared from total RNA and sequences generated on the NovaSeq platform using 150 base pair, pair-end sequencing. Trimmed reads were uploaded on to Galaxy Europe [48] for analysis and mapped to NCBI reference genome idEpiBalt1.1 (accession GCF_945859705.1) [49] using STAR to produce a list of read counts per gene [50]. This list was fed to DESeq2 [51] for estimating differentially expressed genes (DGE), comparing females with male individuals. Differentially expressed genes were filtered by a log2fold change of |1.5| and an adjusted *p*-value of <0.001. The resultant gene list was used to carry out a BLAST search against *Drosophila melanogaster* coding sequences (dmel-all-CDS-r6.55) with hits that demonstrated >50% coverage and an e-value of <0.05 were used to assign *D. melanogaster* gene names to *E. balteatus* sequences. These were then used to elucidate gene functions using Flybase [52] and literature searches.

## 3. Results

### 3.1. Female migrants are more abundant than males in the Pyrenees

**Figure 1.**
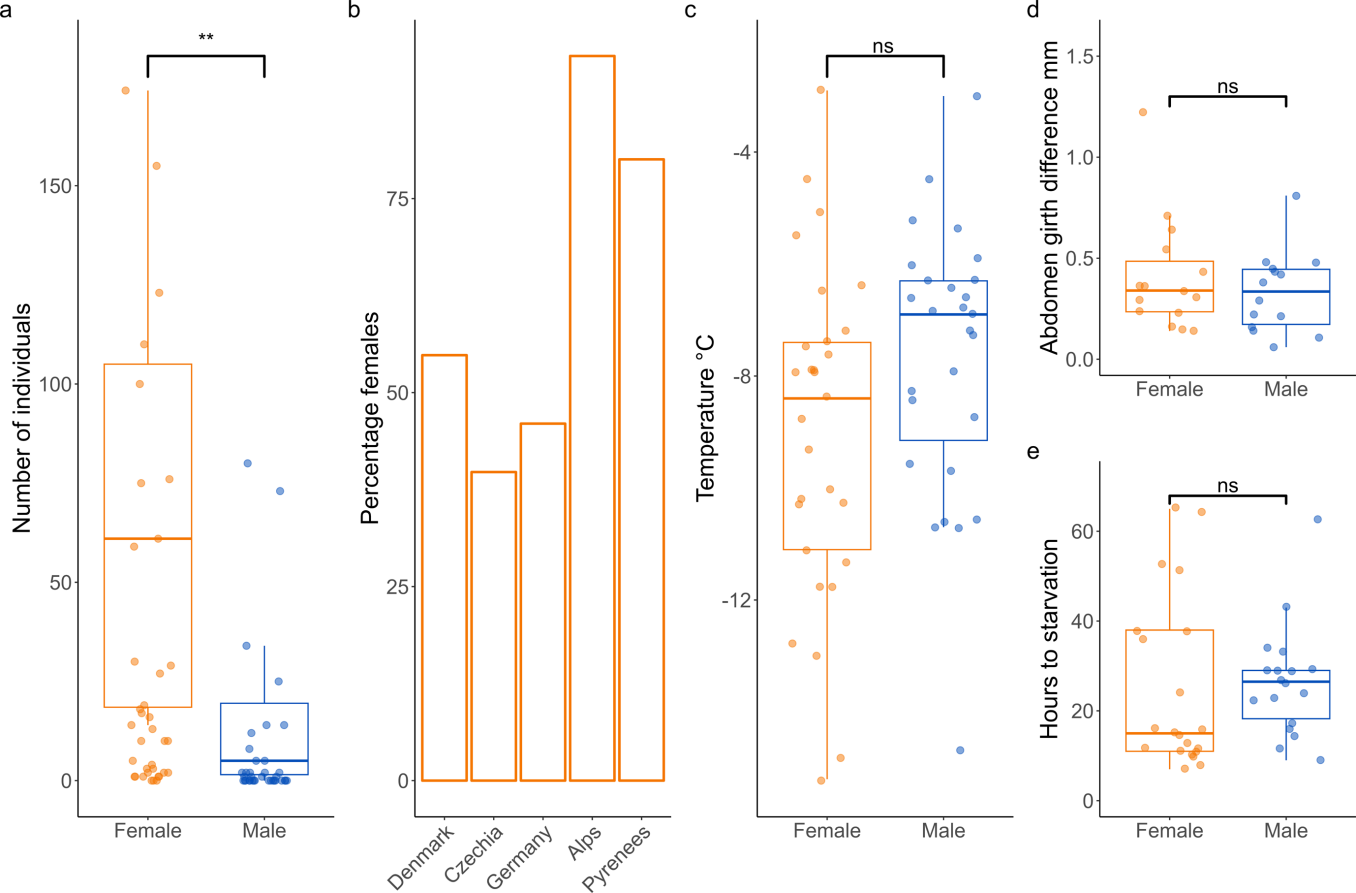
(*a*) Total *E. balteatus* collected per day by malaise trap during 17 days of mass migratory events [19] at the Puerto de Bujaruelo during September and October, 2018−2021. (*b*) Percentage female migratory *E. balteatus* caught heading south in autumn (August–October) at migration bottlenecks in Europe ordered from highest latitude to lowest. Data from Denmark (sweep netting, lat 55.69), *n* = 197 [53], Czechia (malaise trap, lat 50.12), *n* = 1084 [22], Germany (small funnel trap, lat 48.35), *n* = 2713 [33], Swiss Alps (small funnel trap, lat 46.14), *n* = 1569 [54], Pyrenees (malaise trap, lat 42.70), *n* = 821 (this paper). (*c–e*) Physiological experiments conducted on female and male migrant *E. balteatus* in Puerto de Bujaruelo. (*c*) Super cooling point. (d) Abdomen girth following a 48 h feeding period. (*e*) Hours taken until starvation.

Utilizing 4 years of migration malaise trap data, we found that female *E. balteatus* were significantly greater in abundance than males during mass migratory events (Linear model, *F*_28,29_=10.367, *p* = 0.00324; figure 1a) (electronic supplementary material, file S2). Analysis of sex ratio data from across Europe demonstrated an increase in females as flies migrate south during the autumn period (figure 1b) with 54% females in Denmark rising to 93% and 80% in the Alps and Pyrenees respectively (electronic supplementary material, file S2E).

### 3.2. Female migrants show increased cold tolerance but no difference in hyperphagia or starvation resistance

We see a relationship approaching significance in cold tolerance between females and males (linear model, *F*_54,55_ = 3.3809, *p* = 0.0715). However, when a male outlier was removed (−14.9°C) we recovered a significant interaction between sex and cold tolerance (linear model, *F*_53,54_ = 5.3885, *p* = 0.02415; figure 1c) with mean SCP in females at −9°C versus −7.7°C in males, while body condition had no significant effect on SCP. There was no significant difference in fly abdomen girth increase or starvation resistance between male and female migrants (model results respectively; linear model, *F*_30,31_=1.1729, *p* = 0.2682, linear model, *F*_37, 38_ = 0.0933, *p* = 0.7618; figure 1d,e). Raw data can be found in electronic supplementary material, files S3–S5.

### 3.3. Migrants have larger wings than non-migrants

**Figure 2.**
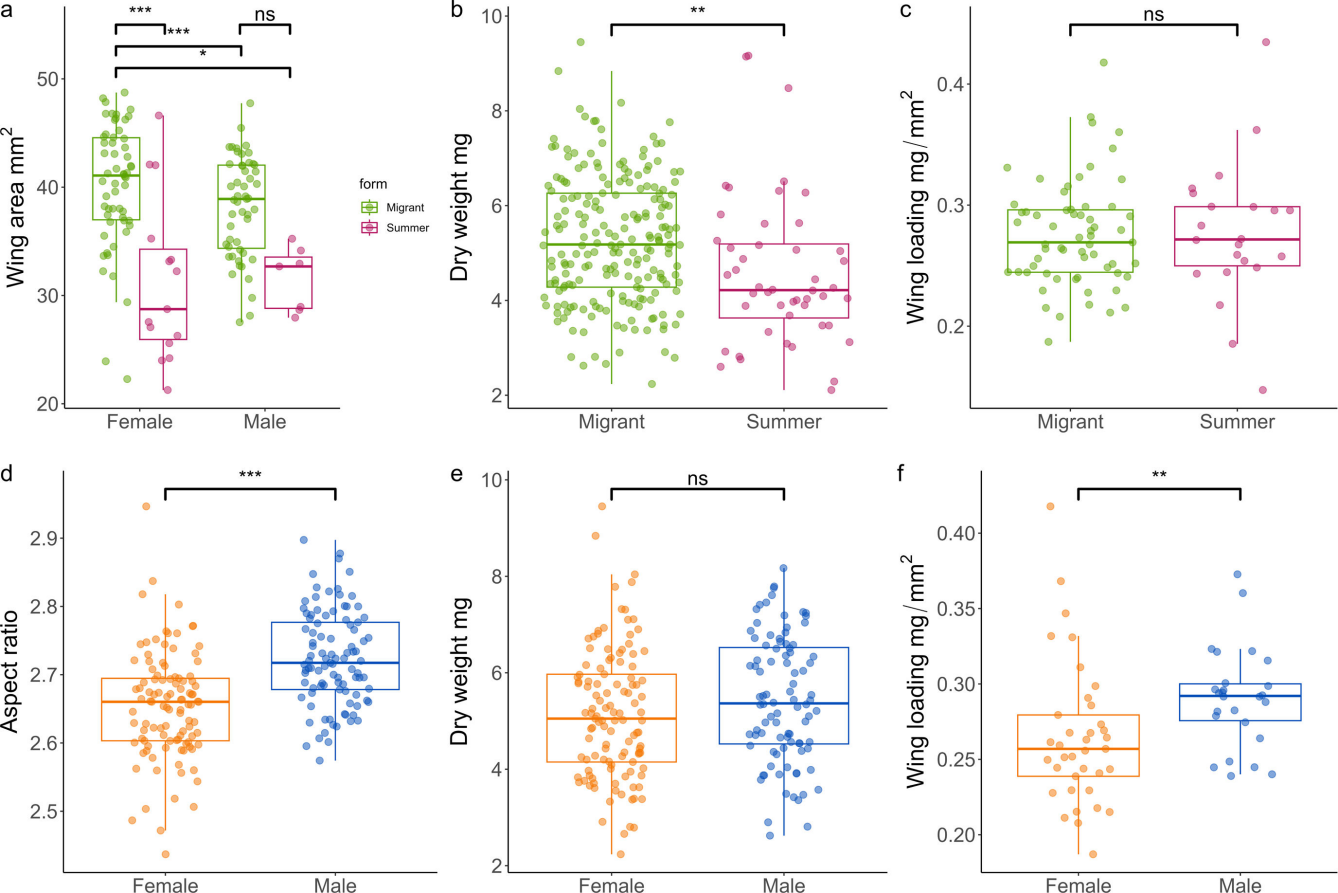
Wing measurements, dry weight and wing loading of *E. balteatus*. (*a*) Wing area comparison between female and male flies with the interaction of form, significance was calculated using the post hoc pairwise comparison *Emmeans*. (*b*) Dry weight of migrant and summer forms. (*c*) Wing loading of migrant and summer forms. (*d*) Wing aspect ratio of female and male migrants. (*e*) Dry weight of female and male migrants. (*f*) Wing loading of female and male migrants.

We compared wing measurements in migrant and non-migrant flies. Migrants showed significantly larger wings than non-migrant summer phenotypes with an interaction of form and sex (wing length; Linear model, *F*_163,164_ = 10.190, *p* = 0.002, wing width; Linear model, *F*_163,164_ = 11.666, *p* < 0.001, wing area; Linear model, *F*_80,81_ = 6.155, *p* = 0.015; figure 2a) and dry weight (a body size proxy) (wing length; Linear model, *F*_163,164_ = 349.84, *p* < 0.001, wing width; Linear model, *F*_163,164_ = 237.984, *p* < 0.001, wing area; Linear model, *F*_80,81_ = 148.2567, *p* < 0.001) predicting wing size. Wing aspect ratio (AR), the ratio between wing length and wing width, reveals summer forms to have a greater aspect ratio (Linear model, form; *F*_159,160_ = 11.316, *p* =<0.001), with additional interactions of dry weight with heavier individuals exhibiting a higher AR (Linear model, dry weight; *F*_159,160_ = 4.694, *p* = 0.032) and sex with males exhibiting a higher AR (Linear model, sex; *F*_159,160_ = 18.610, *p* =<0.001). Dry weight revealed migrants to be significantly heavier than summer individuals (Linear model, *F*_255,256_ = 9.555, *p* = 0.002; figure 2b), but we found no difference in wing loading (dry weight/total wing area) (Linear model, *F*_83,84_ = 2.174, *p* = 0.144; figure 2c).

### 3.4. Female migrants show lower wing loading values

Wing measurement comparisons between migrant females and males showed females had significantly longer wings (linear model, *F*_136,137_ = 18.246, *p* =<0.001); wider wings (linear model, *F*_136,137_ = 37.142, *p* =<0.001) and larger total wing area (linear model, *F*_60,61_ = 19.098, *p* =<0.001; figure 2a). Additionally, dry weight significantly affected these wing size metrics (wing length; linear model, *F*_136,137_ = 292.630, *p* =<0.001, wing width; linear model, F_136,137_ = 184.870, *p* =<0.001, wing area; linear model, *F*_60,61_ = 133.686, *p* =<0.001). The larger wings seen in females however did not translate into a higher AR as they were larger in proportion, whereas we saw a significantly higher AR in males (linear model, *F*_200,201_ = 43.795, *p* =<0.001; figure 2d). Finally, while there was no significant difference in dry weight between sexes (linear model, *F*_207, 208_ = 2.5675, *p* = 0.1106; figure 2e), the increased wing area seen in females did significantly lower wing loading (linear model, *F*_60,61_ = 26.146, *p* =<0.001; figure 2f), as did dry weight, indicating that increased body size results in increased wing loading (linear model, *F*_60,61_ = 220.188, *p* =<0.001).

### 3.5. Females outperform males in measures of flight capacity

**Figure 3.**
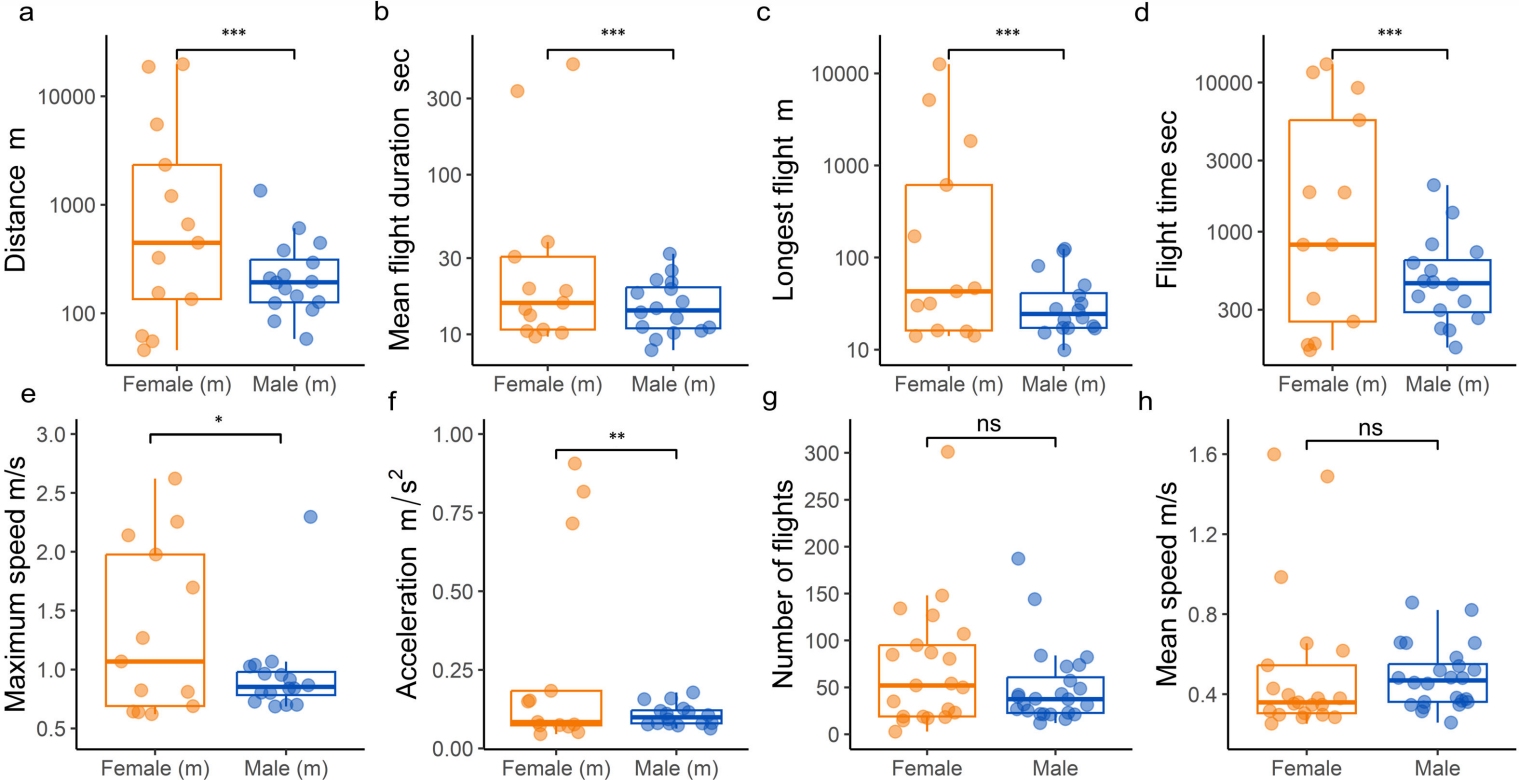
Flight performance of female and male *E. balteatus*. (*a–e*) Medium body condition comparisons denoted by (m). (*g–h*) Comparisons from all body conditions. (*a*) Log total distance flown (metres). (*b*) Log mean flight duration (seconds). (*c*) Log longest distance flown (metres). (*d*) Log flight time (seconds). (*e*) Maximum speed (metres per second). (*f*) Acceleration (metres per second squared). (*g*) Number of flights. (*h*) Maximum speed (metres per second).

Females significantly outperformed males in a variety of flight performance and propensity metrics over 4-h flight periods with body condition representing a significant interaction. Medium sized females flew a greater distance than medium sized males averaging 2566 m (±2275 m) compared with 290 m (±194 m) (GLMM, *β* = 2.177, s.e. = ± 0.490, *z* = 4.447, *p* = 0.0001; figure 3a) and while medium females flew further than fat females (GLMM, *β* = 1.753, s.e. = ± 0.596, *z* = 2.941, *p* = 0.0384), medium males flew similar distances to fat males (GLMM, *β* = 1.071, s.e. = ± 0.732, *z* = 1.463, *p* = 0.6880). This pattern was also found for average flight duration with medium females (59 ± 37 s) exhibiting longer flights than medium males (17 ± 9 s) and fat females (16 ± 13 s) (model results respectively; GLMM, *β* = 1.2545, s.e. = ± 0.364, *z* = 3.450, *p* = 0.0006, figure 3b; GLMM, *β* = 1.2812, s.e. = ± 0.425, *z* = 3.018, *p* = 0.0306). Similarly, the longest flight distance of medium females (934 ± 1241 m) was significantly greater than those of medium males (38 ± 41 m) and fat females (57 ± 82 m) (model results respectively; GLMM, *β* = 3.2164, s.e. = ± 0.607, *z* = 5.297, *p* < 0.0001; figure 3c; GLMM, *β* = 2.7877, s.e. = ± 0.641, *z* = 4.350, *p* = 0.0002). In addition, medium females also spent significantly more time flying than medium males averaging 3134 s (± 2106 s) compared with 572 s (± 300 s) (GLMM, *β* = 1.6977, s.e. = ± 0.387, *z* = 4.382, *p* = 0.0002; figure 3d). Medium females reached higher maximum speeds than medium males (averaging 1.266 m s^−1^ ± 0.335 versus 0.943 m s^−1^ ± 0.233) (GLMM, *β* = 0.294, s.e. = ±0.133, *z* = 2.219, *p* = 0.0265; figure 3e). Lastly, medium females had superior acceleration (0.219 ± 0.097 m s^−2^), displaying rates almost two times greater than medium males (0.112 ± 0.04 m/s^2^) and over three times that of fat females (0.068 ± 0.04 m s^−2^) (model results respectively; GLMM, *β* = 0.667, s.e. = ±0.253, *z* = 2.637, *p* = 0.0084; figure 3f; GLMM, *β* = 1.157, s.e. = ±0.320, *z* = −3.613, *p* = 0.0041). No significant differences were found in number of flights (*F*: 71 ± 24 versus *M*: 50 ± 24) or mean speed (*F*: 0.503 m s^−1^ ± 0.04 versus *M*: 0.483 m s^−1^ ± 0.121) between males and females (model results respectively; GLMM, *β* = −0.3540, s.e. = ± 0.2339, *z* = −1.513, *p* = 0.13; figure 3g; GLMM, *β* = 0.0399, s.e. = ± 0.124, *z* = −0.320, *p* = 0.749; figure 3h). Raw flight mill data can be found in electronic supplementary material, file S6.

### 3.6. Identification of sex-specific migration associated genes

**Figure 4.**
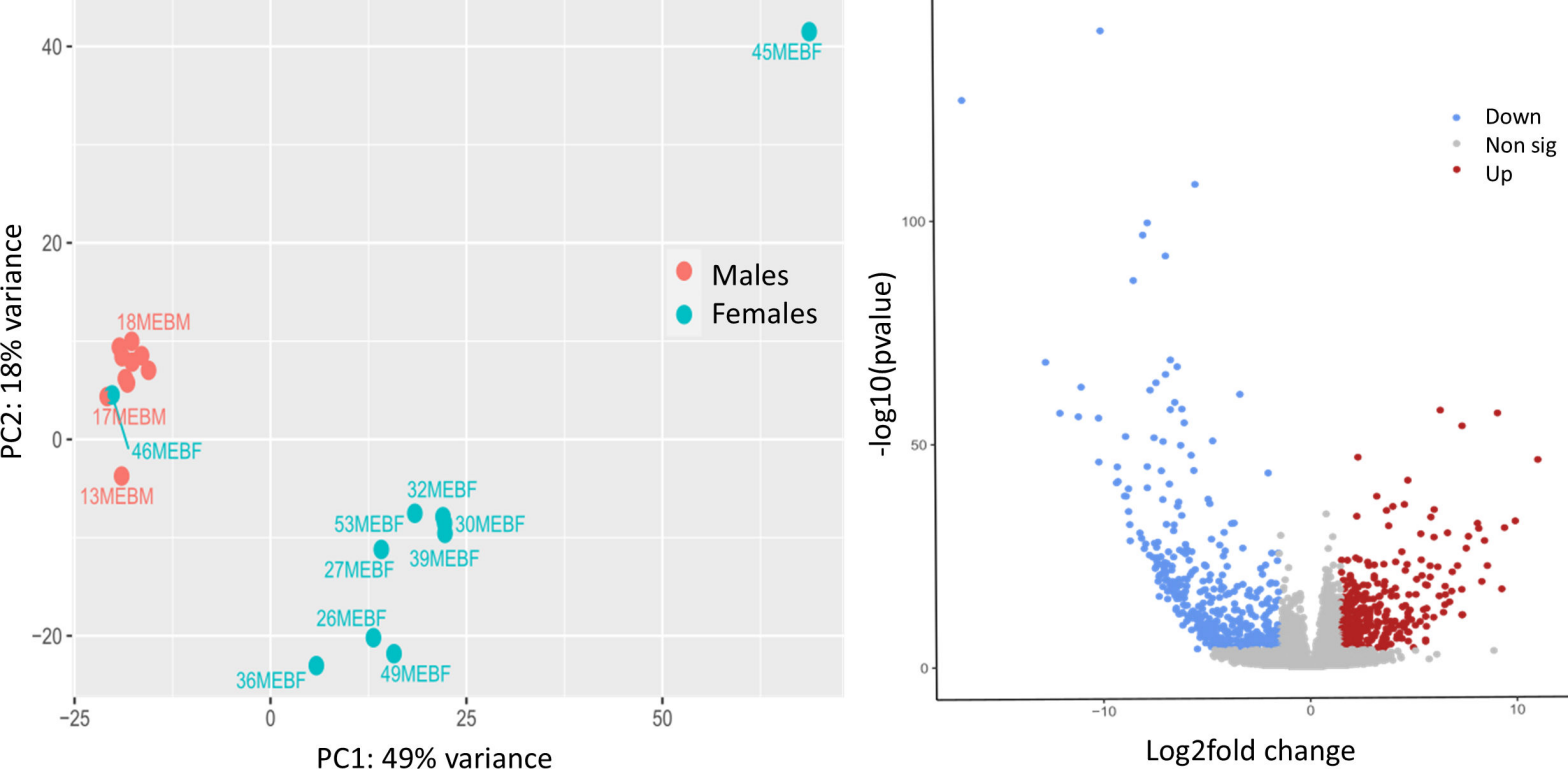
Sample similarities and DGE. (*a*) Principal component analysis plot of all genes expressed in samples from females (turquoise) and males (red); due to tight clustering, male samples 1MEBM, 3MEBM, 4MEBM, 5MEBM, 10MEBM, 11MEBM and 12MEBM are not labelled. (*b*) Volcano plot of downregulated (blue), upregulated (red) and nonsignificant (grey) genes using adjusted *p*-values of <0.001 at and log2fold change of |1.5|.

To identify potential transcripts involved in female migratory success, we compared the genome-wide transcription profiles of actively migrating individuals caught as they traversed the pass southward through the Pyrenees. Total RNA was extracted from 10 whole individuals of each group, obtaining RNA with 260/280 ratios between 1.95 and 2.03 and distinct peaks in Bioanalyzer outputs, samples were then subject to Illumina cDNA library preparation and sequencing. A total of 443 million paired-end 150 bp reads were sequenced with an average yield of 22 million reads per sample. Following quality control, reads were aligned to the annotated *E. balteatus* genome using STAR to produce a list of read counts per gene, the average uniquely mapped reads totalled 18,461,635 or 83.06% (see electronic supplementary material, file S7), DGE was estimated using DESeq2. PCA analysis (figure 4a) of all reads showed distinct signatures for males with two outlier samples in females, one of which tightly clustered with the males. A total of 792 genes out of an estimated total number of 13 663 protein coding genes present in the genome (6%) showed very strong evidence of differential expression with 357 or 45% upregulated and 435 or 55% downregulated (figure 4b; electronic supplementary material, file S8). The terms up- and downregulation are based on differential gene expression of genes from females compared with males. Therefore, upregulation refers to a gene upregulated in females compared with expression in males and vice versa for downregulation.

### 3.7. Differential gene expression between female and male migrants

**Figure 5.**
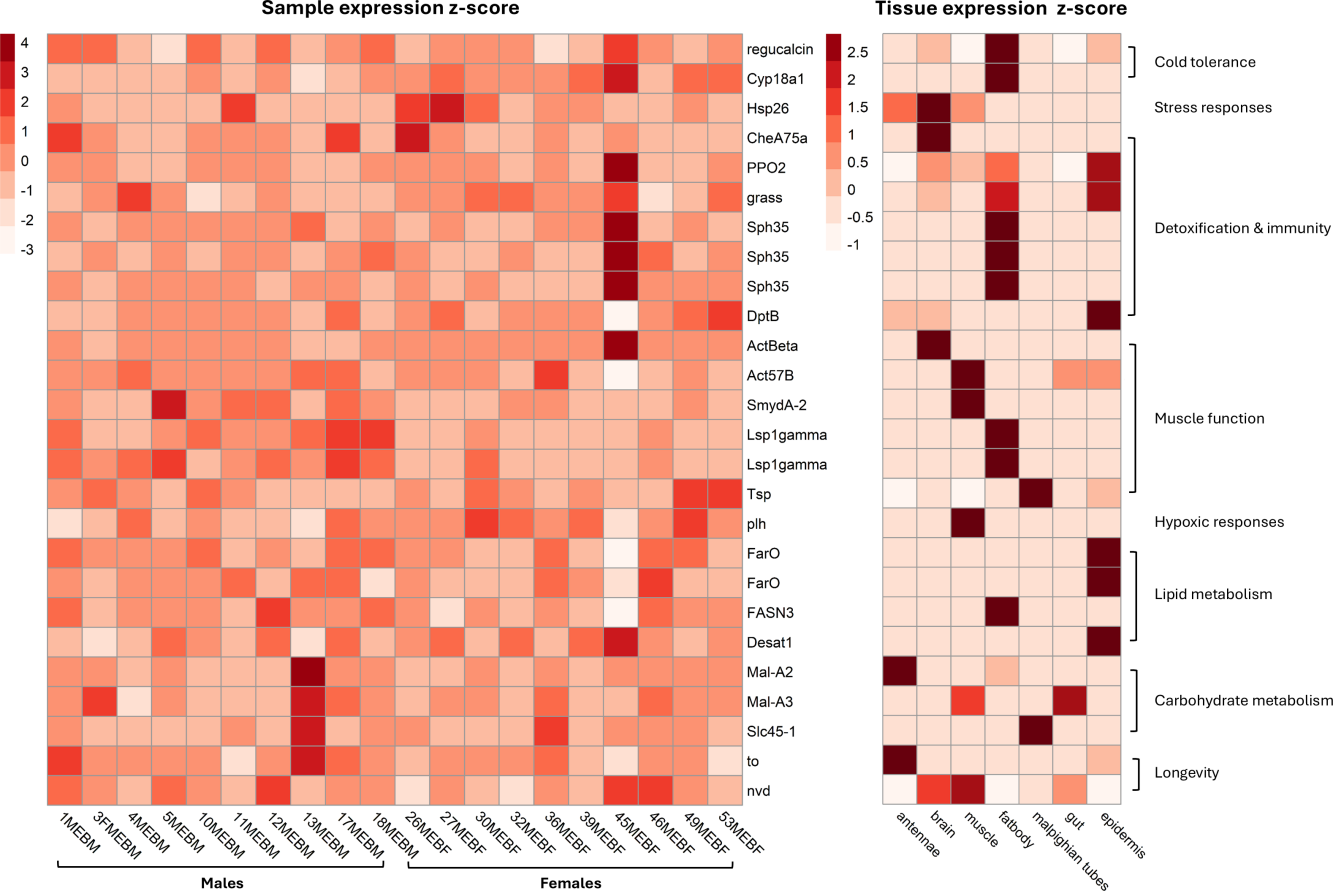
Heat map based on *z*-scores illustrating expression across samples of the key differentially expressed migration-associated genes and tissue-level expression values calculated from samples taken from lab reared summer hoverflies. Key migratory associated genes were determined through extensive literature searches their putative roles in other model organisms.

From this gene set we filtered out reproduction associated genes, using a tissue specific gene expression dataset obtained from the ‘gene expression counts’ output from NCBI RefSeq’s Eukaryote Genome Annotation Pipeline (EGAP) [55,56]. This filtering step removed 186 ovary and 395 testis specific genes, along with 76 genes expressed in both tissues from the complete dataset with (electronic supplementary material, file S9). This resulted in a gene set of 135 differentially expressed genes. A second dataset was generated, post the above-mentioned filtering, removing the outlier female fly sample (46MEBF) that clustered tightly with male fly samples. A PCA was performed with no change in the PCA clustering output (electronic supplementary material, figure S2; results from analysis of this data set can be seen in electronic supplementary material, files S8a–S11a). Of these 135 genes, 67 were also differentially expressed genes in migrant versus non-migrant females, reanalysing data from Doyle *et al*. [6] using the new *E. balteatus* genome assembly [49] and filtering out ovary specific transcripts (see methods and electronic supplementary material, file S10). The resulting dataset consisted of 135 migratory associated genes, 61 were upregulated and 74 were downregulated in female migrants (electronic supplementary material, file S11). The biological processes of these upregulated genes were associated with immunity (6/61), muscle function (6/61), cuticle function (4/61), hormonal processes (3/61), detoxification (3/61), metabolic processes (3/61), life span (1/61), memory (1/48), sensory function (1/61), sexual development (1/61), proteolysis (1/61) and wing development (1/61), along with unknown/uncharacterized functions (31/61). Downregulated genes were positively associated with metabolic processes (12/74), sensory function (6/74), muscle function (4/74), immunity (2/74), detoxification (2/74), life span (1/74), homeostasis (1/74), hormonal processes (1/74), nervous system development (1/74), eye development (1/74) and stress responses (1/74), along with some of unknown/uncharacterized function (41/74). After extensive literature searches, 58 of these 135 genes were identified as having putative roles in migration (see methods and electronic supplementary material, file S12 for references). Here we discuss 26 of these, which we refer to as ‘key genes’, as they exhibit the strongest putative migratory roles, and we explore how they may contribute to migrant success. The differential expression of these genes across samples and tissues is shown in figure 5. For sample and tissue specific *z*-scores (see electronic supplementary material, file S13 and S14, respectively).

## 4. Discussion

Sex biases exist in a variety of taxa due to variation in life history traits and can have a profound influence on migration [36,57]. For instance, heavily female-biased migration of the rice leaf roller *C. medinalis* and the cotton bollworm *H. armigera* occur because of earlier emergence and the need to depart the site of natal origin in search of food and oviposition sites [38,58]. By contrast, monarch butterflies, at least historically, migrated during the autumn and overwinter in roughly equal proportions, yet, in the spring it is the males that emerge earlier [37]. Our data demonstrates a female bias in migratory *E. balteatus* that increases as flies travel south during autumn migration, a pattern in line with data on other hoverfly species in Europe, suggesting this pattern of sex bias is common to other migratory hoverflies [22,33,53,54]. However, this pattern is different from the examples of earlier emergence of insects given above, suggesting different evolutionary drivers may shape the skewed *E. balteatus* sex ratio. One factor that likely underlies this difference is that, while female migrant hoverflies are found in reproductive diapause as in many other migratory insect species, they have additionally already mated and stored sperm within their spermathecae for use in spring when they again become reproductive [32,59]. These observations suggest that males must mate and potentially fight and hold territory during the autumn migratory period, activities that they must trade off against the need for long distance travel but ultimately may increase the risk of degraded tissue and bodily damage. To elucidate the basis of these sex differences, here we present an extensive suite of experimental and transcriptomic data aimed at identifying key differences in actively migrating female and male *E. balteatus* caught during their autumn migration south through the high Pyrenean mountain pass of Puerto de Bujaruelo. This site collects individuals *during* their migratory journey, and while this potentially selects for fitter more efficient flying individuals at higher altitudes, the bias is the same across sexes. Conducting a sampling at the start and/or end of their migratory journey would be advantageous to explore the distribution of traits in starting and successful migrant populations, but this option is severely limited by a lack of sites in Northern and Southern Europe to readily and reliably sample migrants. Equally, comparing migrant and resident males could be advantageous in characterizing the phenotypic changes enabling some males migrate south in autumn. Despite these limitations in sampling, we have demonstrated morphological, behavioural and physiological differences specific to each sex in actively migrating *Episyrphus balteatus* and find that these are supported by a suite of differentially expressed genes which help to explain the female dominated assemblage.

### 4.1. Tolerance to cold and hypoxia in female migrants

Cold tolerance in insects is a plastic trait that varies between and within species, influencing species distribution. In ectotherms, the ability to withstand sudden drops in temperature is a trait allowing survival in a wide range of habitats with several adaptive mechanisms that can be employed such as rapid cold hardening (RCH) and cold acclimation [60–64]. Work by [46] showed that *E. balteatus* is not a highly cold tolerant insect, despite a strong acclimation response when exposed to 0 and −5°C temperatures. While this highlights the importance of migration to warmer climates for the survival of *E. balteatus*, the ability to tolerate extremes of temperature may be also particularly important for long-distance migrants travelling through areas of extreme temperature fluctuations, brought about by high altitude migration [65,66], or migration through mountain passes [19,67] or deserts [21,68]. We find some evidence for a lower SCP in female migrants than males, with values similar to previous studies of non-migrants, suggesting that male migrants are less tolerant of the cold [31,46].

Warmer temperatures are associated with mass-migration events in a range of migrants, including of hoverflies at the Puerto de Bujaruelo [19]. Interestingly, studies have shown female *E. balteatus* exhibit a 2°C lower flight threshold [69] which may enable female flight at lower temperatures, like those found when traversing mountainous regions. To test for this, we looked at how sex ratio varied with temperature but found no trend towards a greater female bias at lower temperatures (electronic supplementary material, figure S3). Regardless, a major candidate in our gene set for a migration-specific cold tolerance factor is the upregulated gene *regucalcin*, encoding a calcium binding protein and linked to cold resistance in *Drosophila* species [70,71]. In addition, we see the upregulation of cytochrome P450 enzyme 18a1 (Cyp18a1), a key gene involved in steroid hormone inactivation and crucial to induce diapause [71–73], that increases stress response mechanisms including cold protein metabolite synthesis in *Drosophila* [74,75]. Finally, we see downregulation of stress induced Heat shock protein 26 (*Hsp26*), suggesting a lower stress status in females [76–78]. Taken together, these findings suggest females may be more tolerant of the cold, and this may provide an advantage as they move through cold regions and undertake high altitude flight (see below).

Hypoxic stress can come from exposure to low oxygen conditions found at altitude, physical exercise or through the combined effects of both altitude and exercise [79]. The latter condition must be addressed by high altitude migrants, for example the bar-headed goose is thought to reduce metabolism to support hypoxic flight during their biannual migration over the Himalayan mountain range [80]. High altitude flight is also common in insects with hoverflies consistently flying at high altitudes over the UK [66], while here we sampled migrants at 2247 m above sea level. Supporting the importance of dealing with hypoxia we detect downregulation of *Pasand Ihamu* (*plh*) in our gene set. The gene (named after the first Nepalese woman to climb the summit of Mount Everest) enhances survival in hypoxic environments following RNAi mediated gene knock downs in *D. melanogaster* [81]. The role of this factor in other high altitude insect migrants remains to be investigated and is complicated by the difficultly in sampling at altitude, except in montane migration bottlenecks that support a high diversity of migrants, such as the Pass of Bujaruelo [19].

### 4.2. Upregulated immunity genes in females

Seasonal migration can profoundly affect the interactions between hosts and pathogens [82]. The effect of migration on immunity has been best studied in birds where it is often associated with immunosuppression, though this may be body condition dependent and rapidly reversed at stopover sites [82–85]. We have previously shown that immunity pathways are modulated during hoverfly migration, and likely function to offset a greater exposure to infection experienced while moving through different geographic areas [6]. A major source of mortality during hoverfly migration can occur via fungal pathogen infection post-migration [21]. Interestingly in this context, we see an up- and downregulation of two transcripts from our gene set that have high similarity to *Chemosensory protein A 75 a* (*CheA75a*) a known chemosensory protein involved in initiation of grooming defence behaviour to fungal pathogens [86]. Along with *CheA75a*, several other differentially expressed genes were identified with roles in immunity, including the upregulation *Prophenoloxidase 2* (*PPO2*), *Gram-positive Specific Serine protease* (*grass*), *Serine protease homolog 35* (*Sph35*) genes and *Diptericin B* (*Dptb*), whose roles involve wound healing and response to bacterial infection [87–89]. This suite of factors suggests a prioritization of immune function in female over male migrants and may demonstrate a greater similarity to the allocation of resources to different branches of the immune system as seen in bats, a group also showing strong sexual differences in migration [90].

### 4.3. Morphological and flight muscle contributions to enhanced female flight performance

Migrant specific changes to morphology that aid long distance movement have been characterized in a range of organisms [91–93]. Our morphological investigations found that migrants are heavier and with a greater wing area when compared with summer individuals. Within migrant populations, females displayed a greater wing area and significantly lower wing loading values than migrant males. Studies in birds have shown that lower wing loading is associated with migration distance [92], while larger forewing area in monarchs has been positively correlated with migration distance to overwintering sites [94]. Our results suggest, as in other migrants, selection is acting on wing size in female hoverflies to increase flight distance and efficiency. Flight performance and propensity are a key trait that differentiate migrants from sedentary populations with a variety of different resources and strategies employed. Within migratory insects, phenotypic changes can be seen in energy utilization, wing morphologies, metabolism and muscle formation [95–99]. Hoverfly flight is driven through powerful flight muscles moving the wings indirectly by deformation of the thoracic exoskeleton, these indirect flight muscles are attached to the exoskeleton via an arrangement of tendons. We tested the flight performance of female and male *E. balteatus*, with body condition (see methods) set as an explanatory variable. We found that, when comparing female and male hoverflies of the same body condition, females exhibited a significantly higher flight performance in terms of distance flown, flight duration, longest flight, time flying and maximum speed. Interestingly, in addition to medium females out competing medium males, medium females also out competed fat females in distance flown, flight duration and longest continuous flight, indicating a trade-off between body condition and flight performance. While medium body condition is a good predictor of flight performance in females, for males this interaction does not improve their flight performance, for instance the longest flight of thin females was significantly greater than the longest flight of medium males. Differences in flight performance seen here may be owing to a variety of factors including lower wing loading seen in females (figure 2*f*).

The role of genetics in flight performance and propensity has only recently started to be unpacked (e.g. [3,4,6]). Our previous study highlighted the role of the transforming growth factor β (TGF-β) signalling pathway in mediating migrant specific muscle adaptations [6]. The TGF-β signalling pathway has two branches initiated by different ligands, activins and bone morphogenetic proteins (BMPs), and is increasingly recognized as an important regulator of many physiological and metabolic processes [100–102]. Doyle *et al*. [6] uncovered a migrant specific promotion of BMPs and repression of activin signalling through downregulation of one of the activin ligands, *Dawdle* [6]. Extending this finding, we detected female specific downregulation of a second activin ligand, *Activin-β* (*Actβ*), supporting the general repression of activin signalling in migrants and suggesting an additional reduction in activin signalling may be occurring in females. How this benefits migrants remains to be tested, but in *D. melanogaster*, Forkhead box O (FoxO) repression of *daw* reduces activin signalling and this alleviates translational repression of *Atg8a*, a key autophagy gene that leads to improvements in muscle performance and life span [103]. Together, these suggest that the slowing of ageing of flight muscle tissue by reduced activin signalling may contribute to flight performance in migrants in general, but particularly in females. By contrast to the maintenance of existing muscle in females, we find evidence for active myogenesis in males based on the expression of *Actin57B* (*Act57B*), a predominant actin gene expressed at all stages of development and the muscle specific transcriptional regulator *SET and MYND domain containing, arthropod-specific, member 2* (*SmydA-2*) [104–107]. Together these data suggest the maintenance of flight muscle in female migrants, while males may rely more heavily on anabolic processes.

In addition to potential muscle specific adaptations, we find evidence for a role in the genes involved in muscle attachment sites. This may be of particular importance as Dipteran flight is driven through flight muscles moving the wings indirectly by deformation of the thoracic exoskeleton, with these powerful indirect flight muscles attached to the exoskeleton via an arrangement of tendons. We find upregulation of muscle attachment factors including *Larval serum protein 1 gamma* (*Lsp1gamma*) and *Thrombospondin* (*Tsp*) both required for muscle attachment stability [108,109]. These attachment points are likely under increased stress during migration owing to the distances being traversed. For example, during spring migration it was estimated that hoverflies flew consistently for a minimum of 200 km during a sea crossing, whereas capture mark recapture experiments have estimated uninterrupted flights of >100 km in 6−12 h [21,25]. In addition to long single flights, hoverflies may traverse thousands of km during the whole autumn migration period [28,110]. Taken together, these findings suggest that the slowing of ageing of flight muscle tissue through reduced activin signalling, along with the maintenance of muscle attachment sites may contribute to the enhanced flight performance seen in female migrants.

### 4.4. Metabolic interactions to sex bias

Insect flight may result in 100-fold or greater increases in oxygen consumption over resting states making fuelling, particularly with energy dense lipids, vital for successful long-distance flight [95,111]. We detect both up- and downregulation of *Fatty acyl-CoA reductase in oenocytes* (*FarO*), implicating its potential importance in both female and male flies. *FarO* promotes lipid droplet induction in oenocytes and may be important for the formation of cuticular hydrocarbons [112,113]. Oenocytes, large secretory cells involved in hepatic and fat body functions including the regulation of energy metabolism, are potentially important for migration as they are thought to process lipids or carbohydrates from the fat body to provide energy under starvation [114]. We also see downregulation in a variety of lipid and carbohydrate metabolic genes: lipids—*FASN3* and *Acsf2* and carbohydrates—*Mal-A2, Mal-A3* and *Slc45-1*. This suggests differences in metabolic strategies between female and male flies with females relying more on lipid metabolism genes (e.g. *Desat1, FarO*) as their primary energy input, whereas males utilize a more diverse array of lipids and carbohydrates. The more diverse mixes seen in males may reflect their mating/migratory strategies that require additional energy, as seen in other insects exhibiting aggressive mating behaviour [115,116].

### 4.5. *takeout* the males

Age modulation is a key adaptative trait in migrant hoverflies allowing vast distances and extended overwintering periods to be covered with reduced age-related mortality [31,117]. A common feature of the migratory syndrome in many insects is reproductive diapause, the cessation of reproduction with resources redirected to other functions such as increased energy stores, resistance to stressors, and in the case of migrants, long-distance flight [118]. Insulin signalling forms one of the key controllers of the diapause programme and has been linked to the suppression of JH and ecdysteroid required to maintain this physiological state [119]. Studies from our lab, and other authors, support the utilization of these pathways in migrants [6,120] although the links between environmental sensing and the various pathways has not been fully elucidated. Highlighting this link, we detect downregulation of the gene *neverland* (*nvd*), encoding an evolutionary conserved oxygenase-like protein required for the biosynthesis of ecdysone [73]. However, the stronger candidate for a central role in the diapause program is *takeout* (*to*), a gene upregulated in our gene set and a circadian clock and FoxO target gene [121] found upregulated in a variety of long-lived flies [122]. For example, in *D. melanogaster*, *to* is a circadian rhythm output gene controlling feeding behaviour and locomotion, with overexpression shown to increase survivorship [123]. Interestingly, a combination of gene knock downs and olfactory behavioural experiments in the migratory locust, *Locusta migratoria*, suggest that the *to* homolog has a role in the formation of locust aggregations [124]. The protein encoding *takeout* contains a JH binding domain which is postulated to bind to JH, blocking signal transduction. This juvenile hormone binding domain is conserved in *E. balteatus to* suggesting a role for this factor in longevity and diapause through IIS signalling and the sequestering of biologically active JH. In summary, our findings suggest a potential role for sex specific upregulation of *takeout* which may shape sex ratios, thus it is plausible that males are ‘taken out’ of the migratory hoverfly population by reduced longevity, or as shown above, through increased susceptibility to pathogens, cold or hypoxia. Future functional studies are needed to address the role of these factors and of *to* in shaping sex ratios of the migratory population.

## 5. Conclusion

Every autumn within the Western European flyway, female and male marmalade hoverflies (*E. balteatus*) undertake a seasonal southward migration to avoid deteriorating environmental conditions. To undertake this journey they undergo a variety of changes to their morphology, physiology and behaviour, yet throughout this southward journey they experience a fundamental transformation in their numbers with females coming to dominate the bioflow, as evident at the Pyrenean pass of Puerto de Bujaruelo. We have shown that a suite of female specific traits are likely to underlie this transformation, including decreased wing loading, specialized metabolism and enhanced flight performance, immunity, longevity and tolerance to cold and hypoxia. These traits ensure the success of female migrants and underlie the diminishing pool of male migrants, influencing population dynamics across huge geographic areas and through the whole migratory and overwintering period. Finally, while we have uncovered a comprehensive picture of sex differences in this species, further work remains to put these findings in a comparative evolutionary framework to understand the commonalities and differences to these strategies and how they affect the seasonal distribution and migratory success of the sexes.

## Data Availability

Trimmed RNA-Seq data and metadata are deposited SRA/NCBI under BioProject ID PRJNA1131956. The genome assembly described in this paper is available using the accession GCF_945859705.1. Tissue-level RNAseq data is available from the Gene Expression Omnibus (GEO) repository via GSE205498. All original code used to process the data is publicly available at [125]. Supplementary material is available online [126].
